# Survival of Patients with Dementia Attending a Psychogeriatric Clinic in Sri Lanka

**DOI:** 10.1192/bjo.2026.11095

**Published:** 2026-06-30

**Authors:** Amodha Medagedara, MGN Priyangani, DVM Dias, Kapila Ranasinghe

**Affiliations:** 1 ELFT, Luton, United Kingdom; 2 Lecturer in Pharmacology, Faculty of Medicine, University of Kelaniya, Ragama, Sri Lanka; 3 NIMH, Angoda, Sri Lanka

## Abstract

**Aims:**

Data on survival rates of patients with dementia is sparse in Asian populations. Thus the aim of this study is to determine rates of survival of patients with dementia in Sri Lanka.

**Methods:**

A descriptive cross-sectional analysis of data was done using the data of patients who have registered at the National Institute of Mental Health Sri Lanka, Older Persons Mental Health Clinic (largest governmental psychogeriatric service provider in Sri Lanka) from 2021 to 2023.

**Results:**

Total registries were 366. Prevalence of Alzheimer’s, vascular and mixed aetiology were 149 (40.41%), 83 (22.67%), 112 (30.6%) respectively. 203 (55.46%) had defaulted up to 2025. Out of 163 under regular follow up 17 (10.43%) had passed away. Mean years of survival with the disease was 3.7 years. Cause of death was dementia and related complications in 13 (76.47%) and vascular events in the rest. Mean age of death had been 77 years and male to female ratio was 0.7. Out of the defaulted 74 (36.45%) had beencontactable. Out of them 57 (77.03%) had passed away. Mean years of survival had been 2.01 years. Cause of death was dementia and related complications in 39 (68.42%) and vascular events in the rest. In the 146 surviving patients under regular follow up mean age was 73. Male to female ratio was 0.55. Mean years of life they had been living with dementia was 4.03 up to 2025. Among 17 surviving contactable patients who had defaulted it was 3.18 years. Odds of death in patients under regular clinic follow up compared with defaulted was 0.035 (Fisher’s exact p-value 2.5´ 10^−^^24^)

**Conclusion:**

Regular clinic follow up is associated with a statistically significant lower rate of death compared to defaulters among patients with dementia.

